# Iliac Vein Compression Syndrome due to Bladder Distention Caused by Urethral Calculi

**DOI:** 10.1155/2015/743270

**Published:** 2015-01-31

**Authors:** Akiko Ikegami, Takeshi Kondo, Tomoko Tsukamoto, Yoshiyuki Ohira, Masatomi Ikusaka

**Affiliations:** Department of General Medicine, Chiba University Hospital, 1-8-1 Inohana, Chuo-ku, Chiba Prefecture, Chiba 260-8677, Japan

## Abstract

We report a rare case of iliac vein compression syndrome caused by urethral calculus. A 71-year-old man had a history of urethral stenosis. He complained of bilateral leg edema and dysuria for 1 week. Physical examination revealed bilateral distention of the superficial epigastric veins, so obstruction of both common iliac veins or the inferior vena cava was suspected. Plain abdominal computed tomography showed a calculus in the pendulous urethra, distention of the bladder (as well as the right renal pelvis and ureter), and compression of the bilateral common iliac veins by the distended bladder. Iliac vein compression syndrome was diagnosed. Bilateral iliac vein compression due to bladder distention (secondary to neurogenic bladder, benign prostatic hyperplasia, or urethral calculus as in this case) is an infrequent cause of acute bilateral leg edema. Detecting distention of the superficial epigastric veins provides a clue for diagnosis of this syndrome.

## 1. Introduction

In patients with iliac vein compression syndrome, iliac venous compression by congenital angiectopia of the iliac artery (May-Thurner syndrome), intrapelvic tumor, aortic aneurysm, retroperitoneal hematoma, retroperitoneal fibrosis, gravid uterus, or another lesion results in edema of one or both lower extremities. In rare cases, this syndrome can be due to a distended bladder. Benign prostatic hyperplasia and neurogenic bladder have previously been reported as underlying causes of bladder distention leading to iliac vein compression, but not the bladder distention secondary to urethral obstruction by a calculus. Here we report a case of iliac vein compression syndrome due to bladder distention caused by a urethral calculus.

## 2. Case Presentation

**Figure 1:**
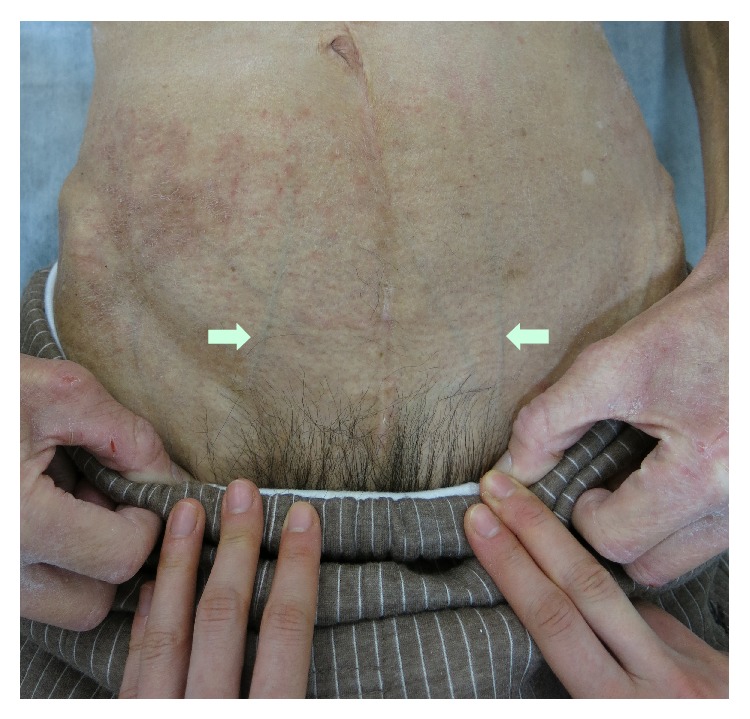
Distention of the bilateral superficial epigastric veins (arrows).

**Figure 2:**
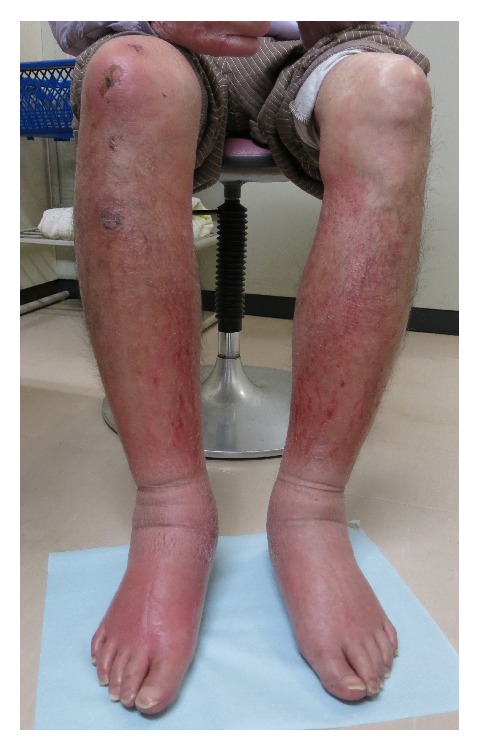
Prominent bilateral lower limb edema with asteatotic eczema.

**Figure 3:**
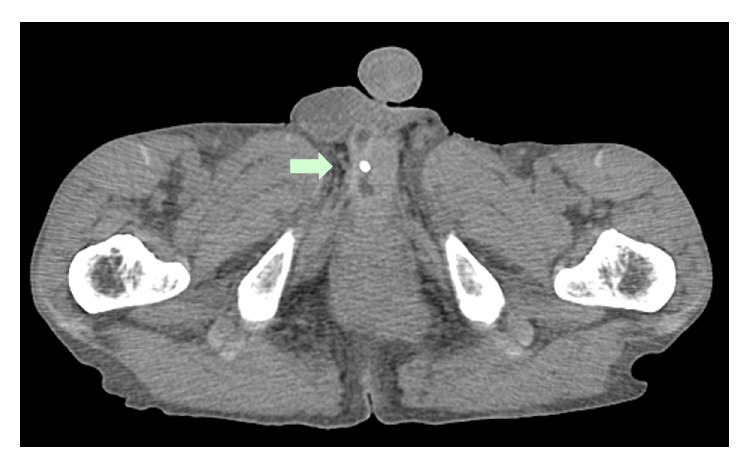
Calculus in the pendulous urethra on plain abdominal CT (arrow).

**Figure 4:**
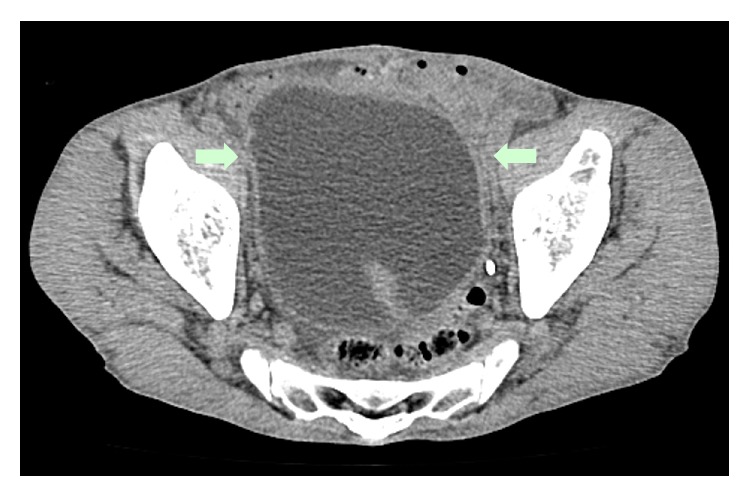
Compression of the bilateral common iliac veins (arrows) by the distended bladder on plain abdominal CT.

A 71-year-old man presented with bilateral leg edema, which had worsened gradually over 1 week along with weight gain of 4 kg. The edema became more severe toward evening, but his legs were not painful. The patient had experienced dysuria for some time, which had become worse around 1 week ago. He had a history of left renal tuberculosis (nonfunctioning kidney) and idiopathic urethral stenosis 20 years ago, as well as asteatotic eczema for several years. He was not on any medications. On physical examination, bilateral distention of the superficial epigastric veins, prominent pitting edema of both lower extremities, and redness of the skin due to asteatotic eczema were detected (Figures 1 and 2). His cardiovascular and lung examination were normal. Because there was bilateral distention of the superficial epigastric veins running to the femoral vein, the patient's bilateral leg edema was believed to be caused by obstruction of the bilateral common iliac veins or else obstruction of the inferior vena cava. Since bilateral leg edema and dysuria had a similar onset, the cause was suspected to be bilateral iliac vein compression by an acutely distended bladder due to retention of urine. Plain abdominal computed tomography (CT) was performed, revealing a calculus in the pendulous urethra (Figure 3), marked distention of the bladder (as well as the right renal pelvis and ureter), and compression of the bilateral common iliac veins by the distended bladder (Figure 4). Based on these findings, iliac vein compression syndrome due to bladder distention caused by urethral calculus was diagnosed. A urethral catheter was inserted to alleviate urinary retention, following which his leg edema gradually improved and resolved after 1 week.

## 3. Discussion

An increase of hydrostatic pressure in the lower limb veins leads to bilateral leg edema and can be caused by volume overload secondary to heart failure or renal failure or rarely by obstruction of the intra-abdominal veins. Iliac vein compression syndrome presents with edema of one or both lower extremities because of venous compression due to congenital angiectopia of the iliac artery (May-Thurner syndrome), intrapelvic tumor, aortic aneurysm, retroperitoneal hematoma, retroperitoneal fibrosis, or gravid uterus among other causes and may be associated with thrombosis at the site of compression. Symptoms are usually chronic and unilateral, but thrombosis can lead to an acute onset and involvement may be bilateral depending on the extent of venous compression. CT scanning and ultrasound are employed for diagnosis. Treatment is directed at the underlying cause of compression and at thrombosis, if present.

In 1960, iliac vein compression syndrome due to bladder distention was first reported by Carlsson and Garsten in a neonate with posterior urethral valves [1]. Since then, adult cases have been reported, chiefly in middle-aged and elderly men. The most common cause is benign prostatic hyperplasia, followed by neurogenic bladder [2]. Iliac vein compression syndrome secondary to bladder distention tends to present with bilateral edema that shows an acute onset, even if it is not associated with thrombosis [2–4]. Accordingly, this syndrome due to bladder distention needs to be considered in patients with acute and bilateral leg edema who do not have heart failure or renal failure. As far as we searched, this is the first report of urinary retention associated with a urethral calculus as the cause of bladder distention leading to bilateral iliac vein compression.

The superficial epigastric vein travels along the abdominal wall and drains into the femoral vein and then into the inferior vena cava via the common iliac vein. Bilateral distention of the superficial epigastric veins indicates development of collateral venous circulation due to obstruction of the common iliac veins or inferior vena cava. Inferior vena caval obstruction with distention of the superficial epigastric veins is known to occur in Budd-Chiari syndrome but has also been identified in past reports of iliac vein compression syndrome [5–7], as well as in the present case. Accordingly, distention of the superficial epigastric vein in a patient with leg edema should raise suspicion of iliac vein compression syndrome.
